# Postoperative analgesia in children when using clonidine in addition to fentanyl with bupivacaine given caudally

**DOI:** 10.11604/pamj.2016.24.182.6446

**Published:** 2016-07-01

**Authors:** Anouar Jarraya, Sahar Elleuch, Jawhar Zouari, Mohamed Smaoui, Sofiene Laabidi, Kamel Kolsi

**Affiliations:** 1 Service d’Anesthésie Réanimation, Hôpital Hedi Chaker, Sfax, Tunisie; 2 Service d’Anesthésie Réanimation, Hôpital Kremlin, Bicêtre

**Keywords:** Caudal epidural, clonidine, fentanyl, pediatric, postoperative analgesia, bupivacaine

## Abstract

The aim of the study was to evaluate the efficacy of clonidine in association with fentanyl as an additive to bupivacaine 0.25% given via single shot caudal epidural in pediatric patients for postoperative pain relief. In the present prospective randomized double blind study, 40 children of ASA-I-II aged 1-5 years scheduled for infraumblical surgical procedures were randomly allocated to two groups to receive either bupivacaine 0.25% (1 ml/kg) with fentanyl 1 μg/kg and clonidine 1μg/kg (group I) or bupivacaine 0.25% (1 ml/kg) with fentanyl 1 μg/kg (group II). Caudal block was performed after the induction of general anesthesia. Postoperatively patients were observed for analgesia, sedation, hemodynamic parameters, and side effects or complications. Both the groups were similar with respect to patient and various block characteristics. Heart rate and blood pressure were not different in 2 groups. Significantly prolonged duration of post-operative analgesia was observed in group I (P<0.05). Side effects such as respiratory depression, vomiting and bradycardia were similar in both groups. The adjunction of clonidine to fentanyl as additives to bupivacaine in single shot caudal epidural in children may provide better and longer analgesia after infraumblical surgical procedures.

## Introduction

In pediatric patients, caudal epidural is commonly used as it is a safe, reliable, and easy method to administer and is therefore the commonly performed procedure for intra-operative and post-operative analgesia especially for sub-umbilical surgeries in young children [1, 2]. One of the main drawbacks of this technique is the short duration of analgesia even with the use of long-acting local anaesthetics like bupivacaine and ropivacaine [3]. The success of achieving prolonged duration of analgesia by the addition of an adjuvant to these local anaesthetics has kept the interest of anaesthesiologists alive for the search of a new adjuvant. Many adjuvants can be used to improve sensory blockade. Epidural fentanyl has been widely used as analgesic adjuvant. It blocks fibers carrying nociceptive impulses in the substantia gelatinosa on the dorsal horn of spinal cord [4]. Clonidine can also be given caudally and it was shown that it exerts analgesic action by stimulating the descending noraderenergic medullospinal pathways and inhibiting the release of nociceptive neurotransmitters in the dorsal horn of spinal cord [5, 6]. Neuraxial administration of clonidine is preferred as it has intense analgesic effect because of its spinal site of action [7, 8]. We conducted this study to assess the efficacy of clonidine in prolonging the action of bupivacaine in association with fentanyl when used for caudal epidural analgesia in children undergoing sub-umbilical surgeries.

## Methods

After obtaining approval from local ethical committee and written informed consent from parents, 40 ASA I (American Society of Anesthesiologists) patients aged 1-5 years, weighing 5-20 kg, scheduled to undergo infraumblical surgical procedures such as hernia repair or orchidopexy were enrolled in this prospective randomized double blind clinically controlled trial. Children with local infection of the caudal area, history of allergic reactions to local anesthetics, coagulopathy, preexisting neurological or spinal diseases, mental retardation, neuromuscular disorders were excluded from the study. Patients were premedicated with hydroxyzine 1 mg/kg given orally 2 hours before surgery. All patients were given general anesthesia. Anesthesia was induced with oxygen, nitrous oxide 50% and sevoflurane 6% with appropriate size face mask and standard monitoring (heart rate, non invasive blood pressure and pulse oximetry). After induction of anesthesia intravenous cannula was placed and I-Gel of appropriate size introduced for ventilation. Anesthesia was maintained with O2-N2O (0.5) and sevoflurane (3%-4%) with assisted respiration with fresh gas flow of 2L/min. Patients were randomly allocated into one of the 2 groups by opening sealed envelope. Group I received bupivacaine 0.25% 1 ml/kg with fentanyl 1μg/kg and clonidine 1μg/kg; while Group II received bupivacaine 0.25% 1 ml/kg with fentanyl 1μg/kg and placebo. Caudal block was given under full asepsis with 23G short bevel hypodermic needle in left lateral position. Patient was turned supine after administration of the drug. The anesthetist in-charge of the patient was completely unaware of the content of syringes. After closure of skin incision, nitrous oxide and sevoflurane were discontinued, the I-Gel was removed and patients were shifted to the post anesthesia care unit (PACU) when fully awake. Heart rate (HR), mean arterial pressure (MAP) and oxygen saturation (SpO2) were recorded before induction of anaesthesia, after induction, before caudal anaesthesia, 10 min after caudal anesthesia and every 10 min thereafter till the patient was shifted to PACU. During intraoperative period adequacy of analgesia was gauged by hemodynamic stability. Absence of rise of HR or MAP of more than 20 % compared with baseline values recorded just before surgical incision was considered as adequate analgesia. An increase in HR or MAP (>20 %), 15 min after administration of caudal anesthesia (at the time of surgical incision) was defined as failure of analgesia. In this case, a dose of 20 μg/Kg of alfentanyl was administrated. Patients, in whom caudal anesthesia failed or inadequate analgesia was present, were excluded from study. Time from caudal block to skin incision, duration of surgery and duration of general anesthesia was recorded as well as demographic parameters (age, weight, size, gender). In post operative period, pain was assessed using CHEOPS Score (Table 1) at H0 (at the admission in the PACU), H1, H2, H4, H6, H12 and H24. CHEOPS score at H12 and H24 was assessed by phone because patients leaved the PACU at H6. In the PACU, if patients had CHEOPS score of >7, or if they requested additional analgesia they were given 0.2 μg/ kg of nalbuphine. After the exit of PACU, all patients were given 15 mg/kg of paracetamol orally 4 times a day. Side effects like motor blockade, nausea, vomiting, respiratory depression, pruritus, hypotension and bradycardia were also noted. Statistical analysis was done using student t-test and chi-square test. P < 0.05 was regarded as statistically significant.

**Table 1:** Children’s Hospital Eastern Ontario Pain Scale (CHEOPS) (recommended for children 1-7 years old) - a score greater than 4 indicates pain

Item	Behavioral	Definition	Score
**Cry**	No cry	1 Child is not crying	
	Moaning	2 Child is not moaning or quietly vocalizing silent cry	
	Crying	2 Child is crying, but the cry is gentle or whimpering	
	Scream	3 child is in a full-lunged cry; sobbing may be scored with complaint or without complaint	
**Facial**	Composed	1 Neutral facial expression	
	Grimace	2 Score only if definite negative facial expression	
	Smiling	0 score only if definite negative facial expression	
**Child verbal**	None	1 Child not talking	
	Other complaints	1 Child complains but not about pain ("I want to see mommy: or "I am thirsty")	
	Pain complaints	2 Child complains about pain	
	Both complaints	2 child complains about pain and about other things (e.g. It hurts; I want mommy.)	
	Positive	0 Child makes any positive statement or talks about other things without complaint	
**Torso**	Neutral	1 Body (not limbs) is at rest; torso is inactive	
	Shifting	2 Body is in motion in a shifting or serpentine fashion	
	Tense	2 Body is arched or rigid	
	Shivering	2 Body is shuddering or shaking involuntarily	
	Upright	2 Child is in a vertical or upright position	
	Restrained	2 Body is restrained	
**Touch**	Not touching	1 Child is not touching or grabbing at wound	
	Reach	2 Child is reaching for but not touching wound	
	Touch	2 Child is gently touching wound or wound area	
	Grab	2 Child is grabbing vigorously at wound	
	Restrained:	2 Child's arms are restrained	
**Legs**	Neutral	1 Legs may be in any position but are relaxed; includes gently swimming or separate-like movements	
	Squirm/ kicking:	2 Definitive uneasy or restless movements in the legs and/or striking out with foot or feet	
	Drawn up/tensed	2 Legs tensed and/or pulled up tightly to body and kept there	
	Standing	2 Standing crouching or kneeling	
	Restrained	2 child's legs are being held down	

## Results

In this prospective randomized study, 40 pediatric patients, 20 in group I and 20 in group II were included. No case of caudal block failure was noted and no patient was excluded. Both the groups were comparable with regard to mean age, weight, gender, duration of general anesthesia, duration of surgery and time from caudal block to incision (Table 2). In the preoperative period, The MAP, HR, and SpO2 were similar for both groups (Table 3). We noted no case of caudal block failure and surgical analgesia in both the groups was found to be adequate. No patient in either group required intraoperative rescue analgesia. No patient in either group required additional analgesia until 6 hours postoperatively. After 6 hours, however, pain score was significantly higher in group II than group I (P < 0.05) (Table 4). The complications and side effects noted were similar in both groups. Residual motor blockade on arrival in PACU was seen in 2 patients in group I and 1 patient in group II. One patient from each group suffered from vomiting. Severe complications such as respiratory depression, bradycardia, urinary retention and pruritus were not observed in this study.

**Table 2:** Demographic parameters

Parameters	Group I	Group II	P value
Age (months)	29.1 ± 2.4	31.2 ± 2.2	0.751
Weight (Kg)	12.7 ± 0.8	13.5 ± 1.1	0.665
Gender (M/F)	18 / 2	20 /0	
Duration of surgery (min)	38.5 ± 6.5	37.1 ± 7.1	0.633
Duration of anesthesia (min)	45 ± 12	42 ± 4	0.398
Time from caudal block to skin incision (min)	9 ± 2	9.5 ± 2	0.273

**Table 3:** Per-operative parameters

-		Ti	T0	T5	T15	T25	T35	Tpo
**HR**	**Group I**	133±23	130±13	126±9	120±12	119±11	127±7	128±4
	**Group II**	124±9	119±14	113±11	113±8	109±12	119±11	127±8
	**P value**	0.140	0.158	0.131	0.337	0.184	0.254	0.88
**MAP**	**Group I**	57 ± 16	56 ± 17	48±11	46±11	47±8	48±9	44±5
	**GroupII**	56 ± 6	60 ± 8	51±8	51±8	50±8	49±9	48±9
	**P value**	0.796	0.198	0.441	0.170	0.199	0.747	0.075

HR: heart rate; MAP: mean arterial pressure;Ti: before the anesthesia induction;T0: at the moment of induction; T5: at the moment of caudal block;T15: 10 minutes after caudal block; T25: 25 minutes after induction; T35: 35 minutes after induction;T po: after the end of the intervention.

**Table 4:** Post-operative CHEOPS Score

-	H0	H1	H2	H4	H6	H12	H24
**Group I**	5.2 ±1.5	4.5±0.5	5.0±0.45	5.5±0.5	5.4±0.7	5.8±1	7.1±0.57
**Group II**	5.8± 0.6	6.3±0.5	5.5± 0.5	6.5±0.5	6.5±0.5	7.1±0.8	7.6±0.52
**P value**	0.288	0.001	0.001	0.001	0.001	0.011	0.091

H0: at the entry in PACU; H1: at the 1^st^ post-operative hour; H2: at the 2^nd^ post-operative hour; H4: at the 4^th^ post-operative hour H6: at the 6^th^ post-operative hour; H12: at the 12^th^ post-operative hour; H24: at the 24^th^ post-operative hour

## Discussion

Caudal epidural anesthesia is a simple, frequently used technique, which provides very effective analgesia intra- and postoperatively in pediatric patients undergoing infraumbilical surgeries. The search for the ideal combination of drugs for caudal anesthesia in pediatric patients is on. Efforts are being made to find relatively safer drugs with minimal side effects. Several adjuvants have been used to prolong the duration of analgesia of bupivacaine for caudal analgesia in children. Opioids, ketamine and midazolam are some of the commonly used drugs [9]. The advantage of clonidine is that it prolongs the duration of analgesia without an increase in the incidence of respiratory depression, pruritus and urinary retention which are commonly seen with neuraxial opioids. Fentanyl, a lipophillic opioid is very commonly used as an additive to local anesthetics in children. Although there is no debate about its beneficial effects, side effects like respiratory depression, pruritus, nausea, and vomiting are common [10]. Clonidine is an alpha-2 adrenoceptor agonist, which was widely used as an anti-hypertensive in 70's and 80's, and presently it has been increasingly used for sedation, premedication, and as an adjuvant analgesic. It is also being used as an adjunct to local anesthetic in neuraxial block. Several mechanisms have been suggested for the clonidine-induced prolongation of caudal analgesia with bupivacaine. The anti-nociceptive action is due to the direct suppression of the spinal cord nociceptive neurons by epidural clonidine. Another mechanism is that clonidine crosses the blood brain barrier and interacts with alpha 2 adrenoceptors at spinal and supraspinal sites to produce analgesia. Clonidine also suppresses neurotransmission in peripheral sensory A δ and C nerve fibres. The final mechanism suggested is pharmacokinetically mediated: clonidine induces vasoconstriction through α-2b adrenoceptors located at the peripheral vascular smooth muscles [11]. The successful use of epidural clonidine in adults led to its evaluation in paediatric caudal epidural block. The resulting studies have consistently shown caudal clonidine to increase the duration of postoperative analgesia [11–15]. The main finding of the present study is that addition of caudal clonidine prolonged analgesia significantly (p<0.05). There was no significant prolongation of motor blockade with addition of clonidine. Hypotension and bradycardia are expected side effect of extradural clonidine in adults and depend on the dose administered, however in children the hemodynamic effects of extradural clonidine are less pronounced than in adults [16]. In the present study, regarding hemodynamics, we did not observe any significant difference in mean heart rate and MAP between the 2 groups, which corroborated to the study result obtained by Laha A et al [15]. No difference was found regarding post-operative sedation between 2 groups, which matched with other study [13, 14]. Parameswari A et al [11] also showed in his study that clonidine in a dose of 1μg/kg, added to 0.25% bupivacaine for caudal analgesia and administered as a 1ml/kg mixture in children, for sub-umbilical surgery, significantly prolongs the duration of post-operative analgesia when compared to 1ml/kg of 0.25% bupivacaine alone, without any side effects. Koul A et al [17] found significant prolongation of post-operative analgesia with an addition of clonidine with bupivacaine caudally. Laha A et al [15] found in a study that the combination of clonidine (2μg/kg) and ropivacaine 0.2% was associated with an improved quality of post-operative analgesia compared to plain 0.2% ropivacaine.

## Conclusion

In conclusion, this study suggests that addition of clonidine (1μg/kg) as an adjuvant with 0.25% bupivacaine and fentanyl through caudal route increases the duration of post-operative analgesia without increasing the incidence of adverse effects.

### What is known about this topic

- The success of achieving prolonged duration of analgesia by the addition of an adjuvant to these local anaesthetics has kept the interest of anaesthesiologists alive for the search of a new adjuvant. Many adjuvants can be used to improve sensory blockade;
- The addition of clonidine (1μg/kg) as an adjuvant with 0.25% bupivacaine through caudal route increases the duration of post-operative analgesia.

### What this study adds

- This study suggests that addition of clonidine (1μg/kg) as an adjuvant with 0.25% bupivacaine and fentanyl through caudal route increases the duration of post-operative analgesia without increasing the incidence of adverse effects.
